# One hundred years of spin: Fundamental physics, dark matter, exotic interactions, and all that

**DOI:** 10.1126/sciadv.aeh6139

**Published:** 2026-09-16

**Authors:** Dmitry Budker, Tim Chupp, Klaus Kirch, W. Mike Snow

**Affiliations:** ^1^ Johannes Gutenberg-Universität Mainz, 55128 Mainz, Germany.; ^2^ Helmholtz Institute Mainz, 55099 Mainz, Germany.; ^3^ GSI Helmholtzzentrum für Schwerionenforschung GmbH, 64291 Darmstadt, Germany.; ^4^ Department of Physics, University of California, Berkeley, Berkeyley, CA 94720, USA.; ^5^ Department of Physics, University of Michigan, Ann Arbor, MI 48104, USA.; ^6^ PSI Center for Neutron and Muon Sciences, 5232 Villigen PSI, Switzerland.; ^7^ Institute for Particle Physics and Astrophysics, ETH-Zürich, 8093 Zürich, Switzerland.; ^8^ Department of Physics, Indiana University, Bloomington, IN 47408, USA.

## Abstract

For a century, spin has been an indispensable probe of the fundamental laws of nature. A reflection on the role of spin in shaping modern physics is presented, from the early days of quantum mechanics to the latest precision tests of the Standard Model. The significance of magnetic and electric dipole moments in testing *CP* and *CPT* symmetries is surveyed, along with the ongoing searches for exotic spin–dependent interactions that may reveal the nature of dark matter and its connection to spacetime geometry. Through these vignettes, it is shown that spin continues to provide a fresh perspective on the most profound questions in physics today.

## INTRODUCTION

### The goals of this paper

The authors of this article are faced with a nearly impossible task: In a few pages, we need to reflect on the connection between spin and fundamental physics. The enormity of this challenge comes from the fact that our modern understanding of nature is impossible to disentangle from spin physics and that these cornerstones of physics as quantum mechanics, quantum field theory, and fundamental symmetries are all intrinsically related to spin. It is for this reason that we decided to present a collection of spin-related “vignettes,” which, without any attempt at completeness, will hopefully give the reader a fresh perspective on the role and importance of spin. (Various complementary aspects of spin physics are discussed by other authors in this collection, for instance, the role of spin in quantum chromodynamics is covered in a review by A. Deshpande, D. E. Kharzeev, and J. Liao.) A key point is that, starting with the early years of quantum mechanics, spin has always played a central role in establishing our fundamental physical picture of the world. This is continuing today, as we illustrate with examples in this article.

### Discovery of spin

Just over 100 years ago, Catalán (*1*) and Landé (*2*, *3*) independently pointed out a puzzling splitting of atomic spectral lines in a magnetic field. An explanation was proposed in 1924 by Pauli (*4*), who introduced the concept of “two-valuedness” inherent to electrons. This idea was further fleshed out by Uhlenbeck and Goudsmit (*5*) in the following year, when they introduced the notion of electron spin. Their bold hypothesis, although initially met with skepticism, would go on to revolutionize and shape our understanding of quantum mechanics. The skepticism is a fascinating topic in itself as it illustrates a clash between naive classical pictures of the microworld and quantum mechanics (*6*). Imagining an electron spin as a classical object with the electron classical radius leads to the conclusion that the surface of the object needs to rotate with a speed two orders of magnitude faster than the speed of light to produce an angular momentum ℏ/2. The discovery of spin not only resolved long-standing puzzles of the time but also opened doors to new technologies, from magnetic resonance imaging (see the articles on magnetic resonance imaging in this collection) to spin-based computing (*7*).

### Proton spin

Proton spin was introduced by Dennison (*8*) in a 1927 paper to explain the specific heat of hydrogen at low temperature, which showed a sharp drop below about 200 K. The discrepancy was resolved by introducing spin 1/2 for the protons and invoking the consequence of the Pauli principle for H_2_. For H_2_, there are two possible proton spin arrangements: total spin *S* = 0 parahydrogen with proton spins opposite and *S* = 1, orthohydrogen, with one and three states, respectively. The wave function of H_2_ is overall symmetric, which is consistent with a rotational ground state *J* = 0 with total electron spin zero and total proton spin zero, i.e., parahydrogen. The energy required for a rotational excitation and transition from parahydrogen to orthohydrogen corresponds to about 200 K. Below that temperature, according to the Boltzmann distribution, the molecules in equilibrium are preferentially in the ground, parahydrogen, state, and the specific heat drops substantially (*8*). Note that, in contrast, deuterium, nuclei are spin-1 bosons, and symmetry requires a symmetric nuclear spin wave function, so the *J* = 0 rotational ground state is “orthodeuterium.” Ortho-para molecular systems also occur with heavier atoms, for example, in ${}^{15}N_{2}$ (*9*).

While on the subject of parahydrogen, it is important to mention that it is not only interesting from the point of view of fundamental physics but is also important in applications as diverse as rocket fuel and nuclear magnetic resonance signal enhancement (*10*) [see also (*9*) for para-${}^{15}N_{2}$].

## SPIN HAVING ITS MOMENTS: MAGNETIC AND ELECTRIC

### Magnetic and electric dipole moments in the Dirac equation

P. Dirac is well known for his work combining quantum mechanics and relativity, resulting in a theoretical prediction of the electron *g*-factor and the interpretation of negative-energy states as antimatter. The equation, which now bears Dirac’s name, was also worked out independently at about the same time by Kramers (*11*): Although his derivation was not published for many years after Dirac’s work appeared (*12*), Kramer’s work was well known to his contemporaries (*13*, *14*).

In Dirac’s 1928 paper “The quantum theory of the electron” (*12*), the Hamiltonian for an electron with spin 1/2 in magnetic and electric fields was derived from a scalar potential $A_{0}$ and a vector potential $\vec{A}$, leading to two terms (note the non-SI, specifically centimeter-gram-second, units)

$$\frac{e\hbar }{2\mathrm{mc}}\left(\vec{\sigma }\cdot \vec{H}\right)+\frac{\mathrm{ie}\hbar }{2\mathrm{mc}}\rho_{1}\left(\vec{\sigma }\cdot \vec{E}\right)$$ (1)

where $\vec{\sigma }$ are 4 by 4 spinor matrices. The first term, the interaction with a magnetic field $\vec{H}$ with the magnetic moment $|\vec{\mu }|=g\frac{e}{2\mathrm{mc}}\frac{\hbar }{2}$, with *g* = 2, agrees with the spinning electron model of Goudsmit and Uhlenbeck (*5*). The second term, the interaction with an electric field $\vec{E}$, includes $i=\sqrt{-1}$. This led Dirac to doubt that it had any physical meaning since the electric field is real. We now understand that this imaginary term changes sign under the time-reversal transformation (*T*) and the $\left(\vec{\sigma }\cdot \vec{E}\right)$ is also odd under parity transformations (*P*). The “electric moment” is therefore *T* and *P* odd, and because of *CPT* invariance (*C* is charge conjugation), it is also *CP* violating. We further discuss electric dipole moments (EDMs) in the “Electric Dipole Moments” section.

### Magnetic moments and spin

Dirac revealed that the magnetic moment of the electron with spin ℏ/2 should be proportional to a factor *g* = 2. Measurements of zero-field splittings in hydrogen and deuterium by Rabi and colleagues (*15*) and Nafe and Nelson (*16*) hinted that g≠2. Radio frequency (RF) spectra of excited-state *n* = 2 hydrogen by Lamb and Retherford (*17*) were also inconsistent with calculations based on Dirac theory. Precise measurements of the hyperfine spectra of Ga and Na by Kush and Foley (*18*) showed results consistent with *g_l_* = 1 and g=2⋅(1.00119) with uncertainties of 50 parts per million (ppm). The corrections to the electron *g*-factor were explained by quantum field theory of electromagnetic interactions or quantum electrodynamics (QED), developed by Schwinger, Feynman, Tomonaga, and Dyson, followed by many others (*19*).

**Fig. 1.**
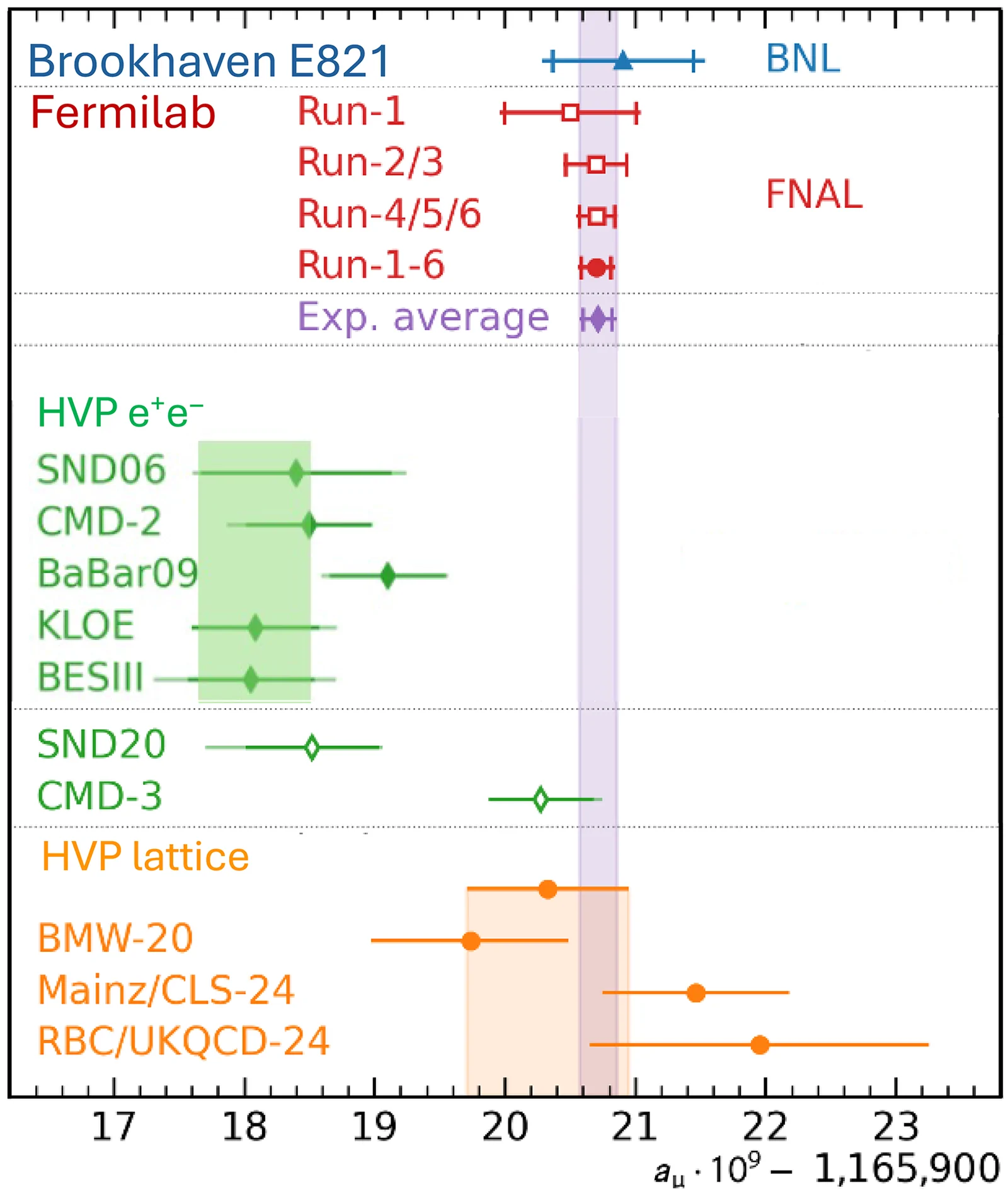
History of muon *g –* 2. Measurements and Standard Model calculations based on semiempirical (green) and lattice QCD (orange) hadronic vacuum polarization contributions. The green and orange boxes represent the Theory Initiative recommendations from 2020 (*22*) and 2025 (*23*).

Over decades, the magnetic moment anomaly, generally expressed as a=(g−2)/2 or just *g –* 2, of the electron and muon have been calculated with increasing precision. The largest corrections, due to QED, have been calculated to fifth order in the fine-structure constant α (*20*). Electroweak (EW) corrections due to the exchange of massive vector bosons for the electron and muon contribute to *g –* 2 at the level of 1.3 ppm and have been calculated with a precision of less than 1% or 10 parts per billion (ppb) (*21*). Both QED and EW correction calculations are perturbative, which is the expansion to higher-order converges. In contrast, strong interaction contributions, specifically hadronic vacuum polarization loops with $\pi^{+}-\pi^{-}$ have presented the greatest challenge and largest uncertainties. Note that the vacuum polarization loop contributions from heavier particle pairs ($\mu^{+}-\mu^{-}$ and $\pi^{+}-\pi^{-}$) scale as the particle mass, and the contributions and the uncertainties are much larger for the muon than for the electron. A dispersion relation approach based on the optical theorem that relates the cross section for $e^{+}-e^{-}$ → $\pi^{+}-\pi^{-}$ was considered the most reliable calculation until recently (*22*). Lattice QCD methods have been applied to calculate hadronic vacuum polarization and the hadronic light-by-light contribution (*23*). The current muon *g –* 2 theory is summarized in the bottom part of Fig. 1 and discussed further in the “Muon *g* − 2” section.

Hadronic vacuum polarization contributions dominate the uncertainty of the Standard Model calculation of the muon magnetic moment anomaly. In contrast, these contributions are small compared to the experimental precision on the electron’s anomaly but may soon become significant with anticipated improved measurement of the electron anomaly.

Precise measurements of the magnetic moment of the electron and, later, the muon became a testing ground for the power of quantum field theory to precisely calculate their interaction with the quantum vacuum. These interactions encompass all of the Standard Model particles and interactions as well as potential beyond the Standard Model (BSM) interactions, and tensions between measurements and Standard Model calculations have motivated recent and prospective experimental efforts discussed below.

**Fig. 2.**
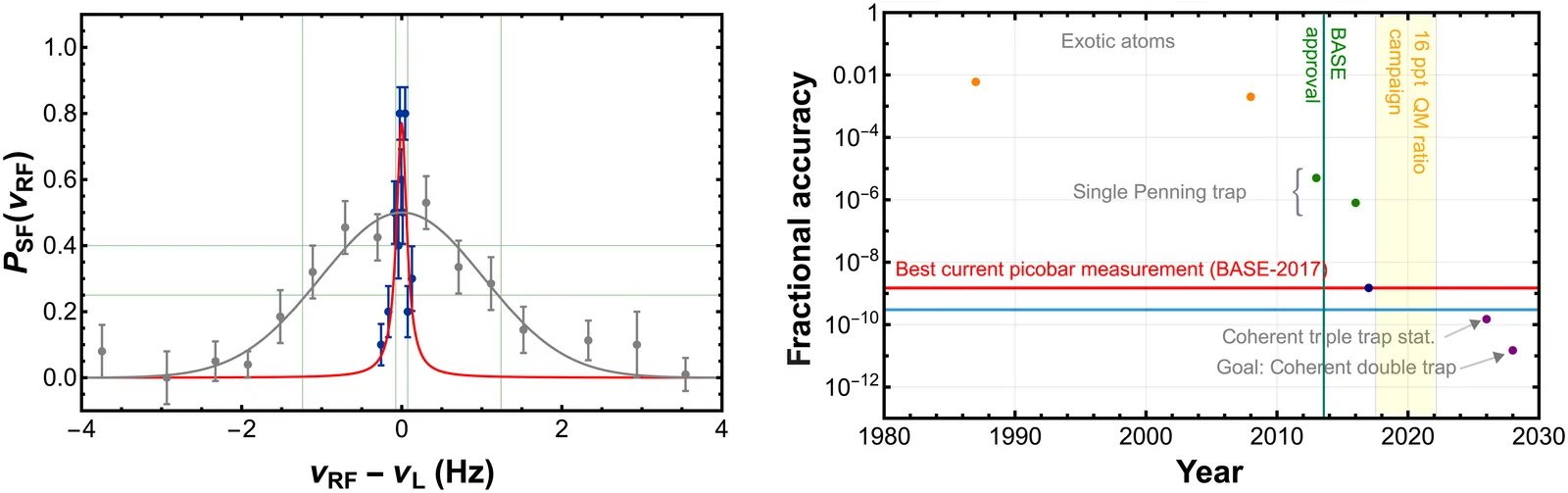
Magnetic resonance of a single antiproton spin and fractional resolution achieved in measurements of the antiproton magnetic moment as a function of time. Left: The plot shows the most recent results from the BASE collaboration [resonance width of 0.156(4)-Hz full width at half maximum (FWHM), blue points] and the least-squares fit of a Voigt profile to the data (red line). The gray data points and fit are from the previously measured antiproton resonance with a width of 2.5(2)-Hz FWHM (*112*, *113*). Right: A fractional accuracy on the 10^−3^ level was reached with exotic atom spectroscopy (orange); measurements in single Penning traps achieved resolutions at the part-per-million level. Upon approval, the BASE collaboration measured the antiproton magnetic moment with a fractional resolution at the 10^−9^ level. It is expected that recently introduced coherent spectroscopy will allow for triple-trap measurements at the level of ≈0.1 ppb. BASE is currently developing a single-particle, double-trap technique with cyclotron frequency measurements based on phase sensitive detection, with a projected (not yet measured) fractional resolution of <20 ppt. Figure is courtesy of S. Ulmer (BASE).

Symmetry under *CPT*, the combined discrete transformations of charge conjugation, parity, and time reversal, is a fundamental principle underlying any quantum field theory (*24*). *CPT* symmetry requires that the magnetic moments of a particle and its antiparticle are equal in magnitude but opposite in direction with respect to the angular momentum $\vec{J}$. Measurements of magnetic dipole moments of the electron and positron, negative and positive muons, and proton and antiproton (see Fig. 2) all confirm *CPT* symmetry. Measurement of the proton and antiproton EDMs (see the “Reasons for measuring EDMs” section), in principle possible in the same storage ring (*25*), would provide a *CPT* test of a *CP*-violating observable.

### Direct measurements of the electron *g*-factor

By the early 1950s experiments with electrons bound in atoms had established and QED had calculated, corrections to the the electron magnetic moment *g*-factor. Measurement of unbound (free) electrons, for example, using the Stern-Gerlach approach was considered with a long debate about the limitations imposed by the uncertainty principle for position and momentum of the electron, see, for example, (*26*). H. R. Crane and students (*27*) pioneered adapting Rabi’s magnetic resonance method to an electron-beam Mott double scattering for electron polarimetry. Their subsequent experiments used a magnetic bottle to essentially trap the electrons for up to about 2 ms with the most precise result for the electron *g*-factor $g_{e}$ with an uncertainty of 27 ppb on $g_{e}$ and 23 ppm on $a_{e}$ (*28*).

Dehmelt and collaborators (*29*) developed measurements with a single or a few electrons in a Penning trap, which used a magnetic field to radially confine the electrons in cyclotron orbits and a quadrupole electric field to confine the electrons along the magnetic field axis. This system of electrons bound by electromagnetic fields to measure the *g*-factor was called “geonium.” The electron motion was detected by measuring the image charge current induced on the electrodes, which also served to damp the motion or cool the energy of the electron motion by coupling the current to a cooled resistor. Several stages of improvement provided the result with an uncertainty of 4 parts per trillion (ppt) on $g_{e}$ and 2.7 ppb on $a_{e}$ (*30*).

Gabrielse and collaborators (*31*–*33*) undertook a series of increasingly precise measurements with a single electron in a cylindrical Penning trap. Improvements over two decades include inhibiting spontaneous emission and cooling the electron to the cyclotron quantum ground state. The currently most precise result is 0.11 ppb uncertainty on $a_{e}$ or 0.13 ppt on $g_{e}$ (*33*).

The Standard Model calculation of $g_{e}$ uses the measured fine-structure constant α as an input. Currently, α is most precisely determined from the recoil energy from photon emission by cesium (*34*) or rubidium (*35*) atoms. The precision on α from these measurements is 200 and 81 ppt, respectively, but they disagree by about 5.5σ. Splitting the difference, this can be incorporated into agreement of the measurement and Standard Model calculation with an uncertainty of 0.7 ppt. This agreement can be interpreted as constraining the electron charge radius to less than about $3\times 10^{-19}$ m and setting limits on the scale of electron substructure (*33*). Alternatively, measurement of $g_{e}$ can be used to determine α, which would fall between the cesium and rubidium values with an uncertainty of 111 ppt.

Improvements to the experimental determination of $g_{e}$ may soon reach a level at which the hadronic contributions for the electron are significant. This would introduce a new challenge to the Standard Model calculations to complement the muon *g*-factor discussed in the “Muon *g* − 2” section.

We conclude this section by highlighting the tremendous progress made in measuring bound-electron *g*-factors using highly charged ions in Penning traps. Recent developments, for example, with H-, Li-, and B-like ^118^Sn ions (*36*–*38*), provide sub–part per billion measurements, challenge higher-order bound-state QED and nuclear theory, and might eventually also lead to an independent extraction of α.

### Muon *g* − 2

Muon magnetic moment anomaly measurements, in part motivated by historical tensions between QED and electron *g*-factor measurements, began in the late 1950s at Brookhaven (*39*) and early 1960s at CERN (*40*). Magnetic storage ring efforts began at CERN, and then a major effort through the 1990s was mounted also at Brookhaven. A decade later, the Brookhaven magnet was rebuilt, and an experiment based on the same concepts with new instrumentation was undertaken at Fermilab. The combined precision on $g_{\mu }-2$, dominated by the past 3 years of data from Fermilab, is 124 ppb (*41*).

Accelerator muons resulting from pion decay are polarized, that is, the spin is strongly correlated with the momentum. The magnetic moment anomaly $g_{\mu }-2$ can be measured with polarized muons injected into a magnetic storage ring, which confines the muons radially. In analogy to the Penning trap, quadrupole electric fields are required for confinement in the third dimension. For relativistic muons, the magnetic moment coupling to magnetic ($\vec{B}$) and electric ($\vec{E}$) fields leads to precession of the spin with respect to the momentum given by the vector anomaly frequency

$$\begin{array}{c} {\vec{\omega }}_{\;a}={\vec{\omega }}_{\;s}-{\vec{\omega }}_{\;c}=a_{\mu }\frac{e}{m_{\mu }}\vec{B}+\frac{e}{m_{\mu }}\left[\frac{a_{\mu }\left(\gamma^{2}-1\right)-1}{\gamma^{2}-1}\frac{\vec{\beta }\times \vec{E}}{c}\right]\\ +\frac{e}{m_{\mu }}\frac{\gamma }{\gamma +1}\vec{\beta }\left(\vec{\beta }\cdot \vec{B}\right) \end{array}$$ (2)

Here, ${\vec{\omega }}_{\;s}$ is the spin precession angular frequency, ${\vec{\omega }}_{\;c}$ is the cyclotron frequency, γ is the relativistic Lorentz factor, $\vec{\beta }$ is the muon velocity divided by the speed of light, and *e* and $m_{\mu }$ are the charge and mass of the muon, respectively. This is the Bargmann-Michel-Telegedi equation (*42*) as rewritten, for example, in (*43*). The first term in Eq. 2 is used to measure $a_{\mu }$ and requires determining $\omega_{a}$ and the magnetic field averaged by the muons over space and time with absolute calibration (*44*, *45*). The second term would lead to significant systematic errors due to the electric field; however, the term can be approximately canceled up to small corrections for γ≈29.3, corresponding to what is called the magic energy. The third term proportional to the muon velocity component along $\vec{B}$ also gives rise to small corrections.

**Fig. 3.**
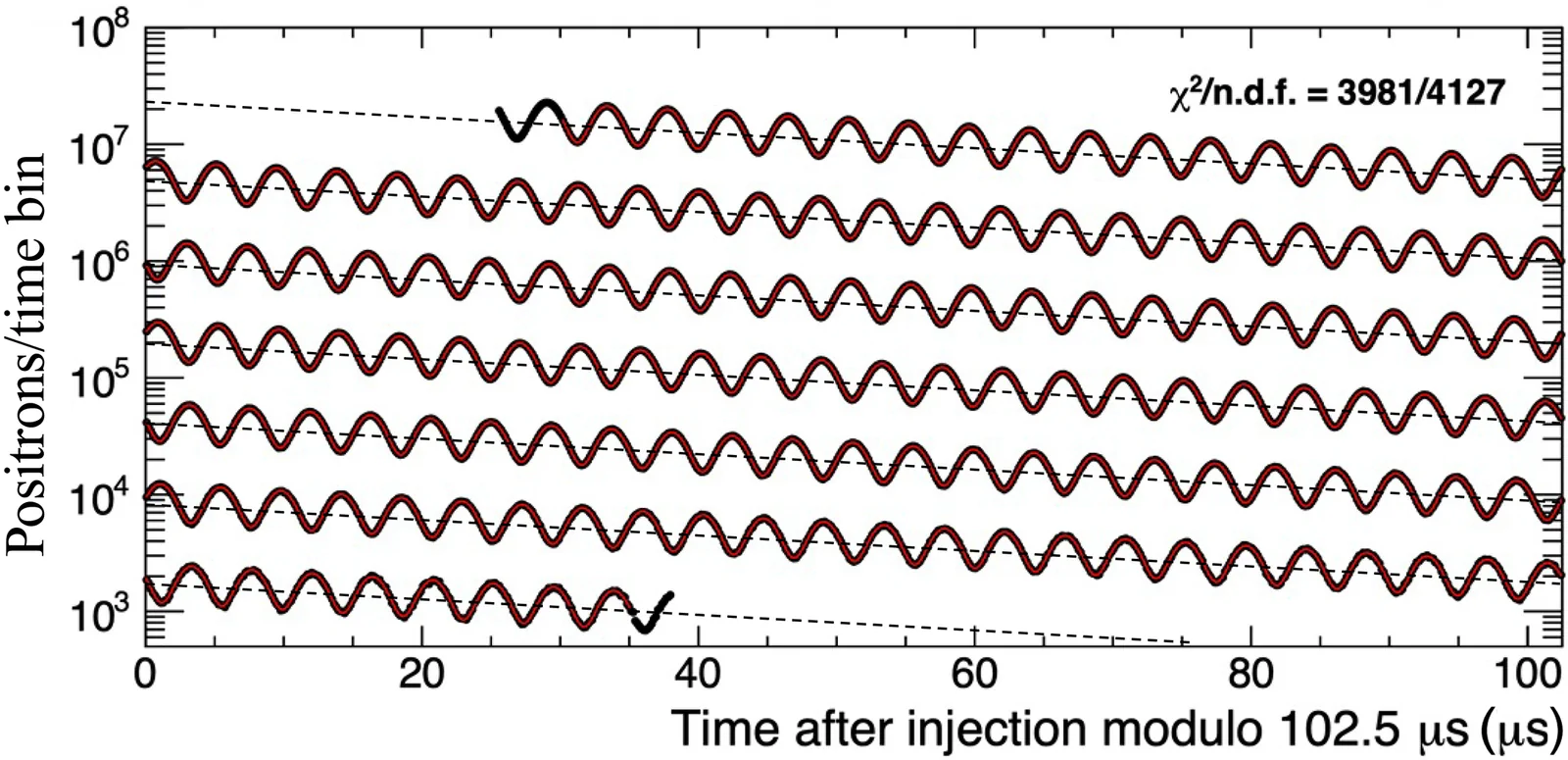
Example of a wiggle plot. Detected, analyzed positrons above a fixed energy threshold are shown as a function of time after injection. The top curve corresponds to 0 to 102.5 μs, the second 102.5 to 205.0 μs, etc. Dashed lines indicate the decay of muons in the laboratory frame. The solid black and red lines are data and a fit from which the muon precession frequency is determined. Figure is based on results from the Muon *g* − 2 collaboration reported in (*114*).

The energy of decay positrons (for $\mu^{+}$) in the laboratory frame is correlated with the relative orientation of the muon spin and decay positron momentum due to parity violation. Calorimeters detect the positrons above an optimum energy threshold resulting in the “wiggle plot” shown in Fig. 3, a truly beautiful demonstration of many modern physics concepts. For example, note that exponential decay trend of muon counts—linear on the semi–log scale—has time constant of about 60 μs, the laboratory frame lifetime ${\gamma \tau }_{\mu }$ (with $\tau_{\mu }=2.2\;\mu s$) due to relativistic time dilation.

The history of measurements at Brookhaven and Fermilab and the Standard Model calculations of $a_{\mu }$ are presented in Fig. 1. The experimental results are notably consistent as the statistics-limited uncertainties have improved by a factor of 4. The Theory Initiative’s estimated uncertainty of the Standard Model calculation has increased to account for disparate semiempirical hadronic vacuum polarization results with recent CMD3 results and the tension of the semiempirical and lattice QCD calculations [see (*23*)]. We anticipate that these estimated uncertainties will improve over time.

The magic energy storage ring approach was statistics limited, and the recently commissioned PIP-II linac at Fermilab and improved storage ring efficiency could conceivably provide a factor of 10 greater detected muon decay rates. A significant improvement would also require reducing the dominant systematic effects, roughly balanced between the magnetic field (mapping, tracking, and calibration) and beam dynamics effects, partly from the second and third terms in Eq. 2.

A new approach to measuring muon *g –* 2 experiment with muonium (the $\mu^{+}-e^{-}$ atom) is planned at JPARC (*46*). Although the precision of about 450 ppb, similar to the combined Brookhaven results, is anticipated, significantly different systematic effects are expected with a factor of 10 lower muon momentum, much smaller emittance eliminating the need for confining electric fields, and a factor of 20 smaller dimension magnet that is much more precise and stable.

## ELECTRIC DIPOLE MOMENTS

### EDM history

In 1950, Purcell and Ramsey (*47*) discussed the “Possibility of electric dipole moments for elementary particles and nuclei.” Mostly referring to the belief in parity conservation, they argued that “suggestive theoretical symmetry arguments” must indeed be tested experimentally. They gave a first estimate for a limiting size of the EDM of the neutron, deduced from scattering experiments investigating the neutron-electron interaction. They thanked their student, Smith, “for suggesting an important correction to” their “original calculation on the neutron-electron interaction experiment.” They also mention that they were undertaking a beam experiment with Smith to directly measure the neutron EDM, essentially using Ramsey’s novel magnetic resonance method of separated oscillatory fields (*48*, *49*) for which he was subsequently awarded the Nobel Prize in 1989.

**Fig. 4.**
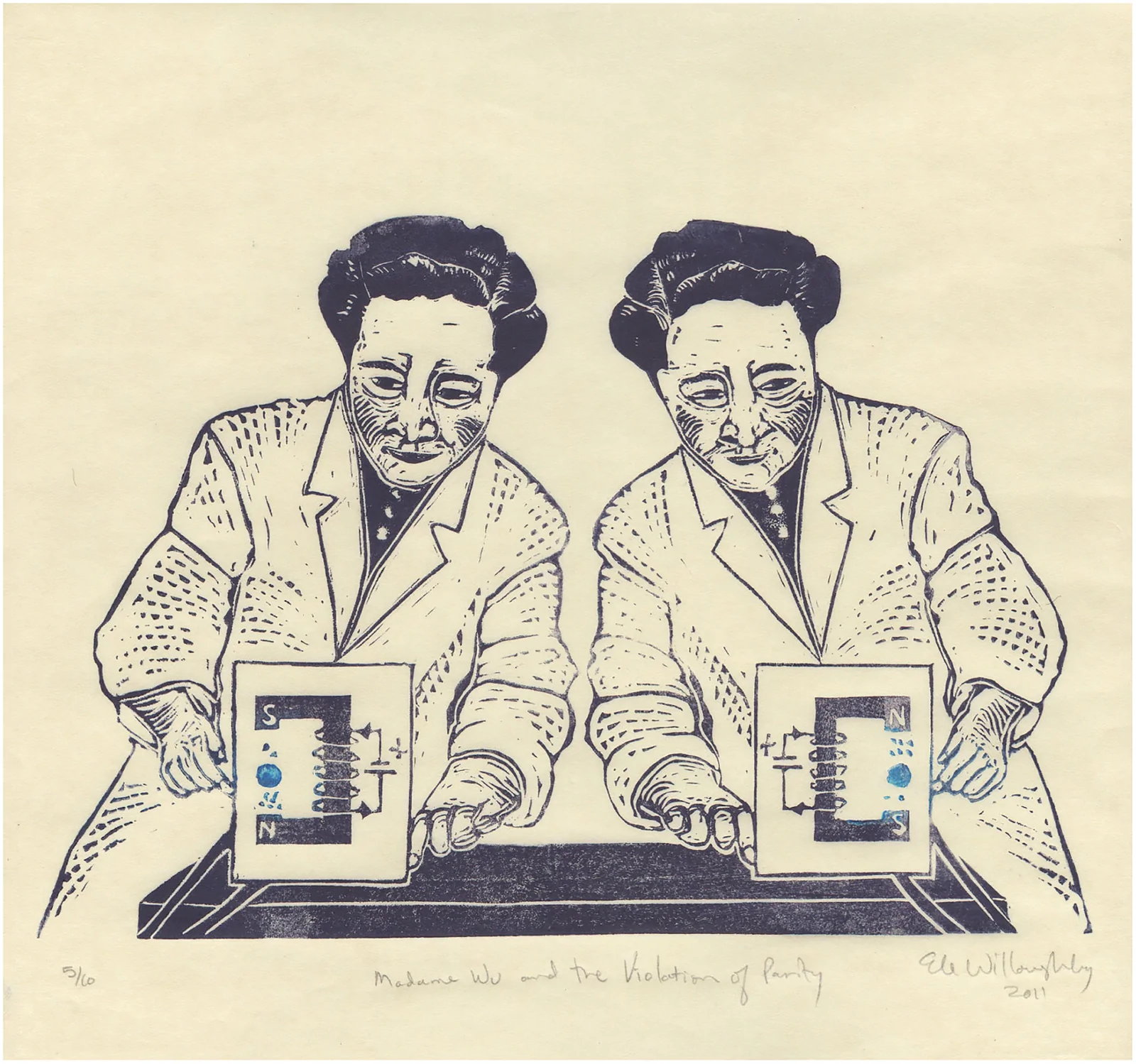
The discoverer of parity violation, C. S. Wu and her famous experiment. The figure shows and image of a lino print by E. Willoughby (reproduced with the artist’s permission). On the left, Madame Wu is depicted with her apparatus where β decay of polarized ^60^Co nuclei (the blue ball) was studied (*50*), in which it was shown that β decay electrons are preferentially emitted in the direction opposite to the direction of the nuclear spin. This is significant because in a “mirror image” experiment (right), one would find that β electrons are preferentially emitted along the spin, which is not observed, signifying left-right asymmetry of nature.

**Fig. 5.**
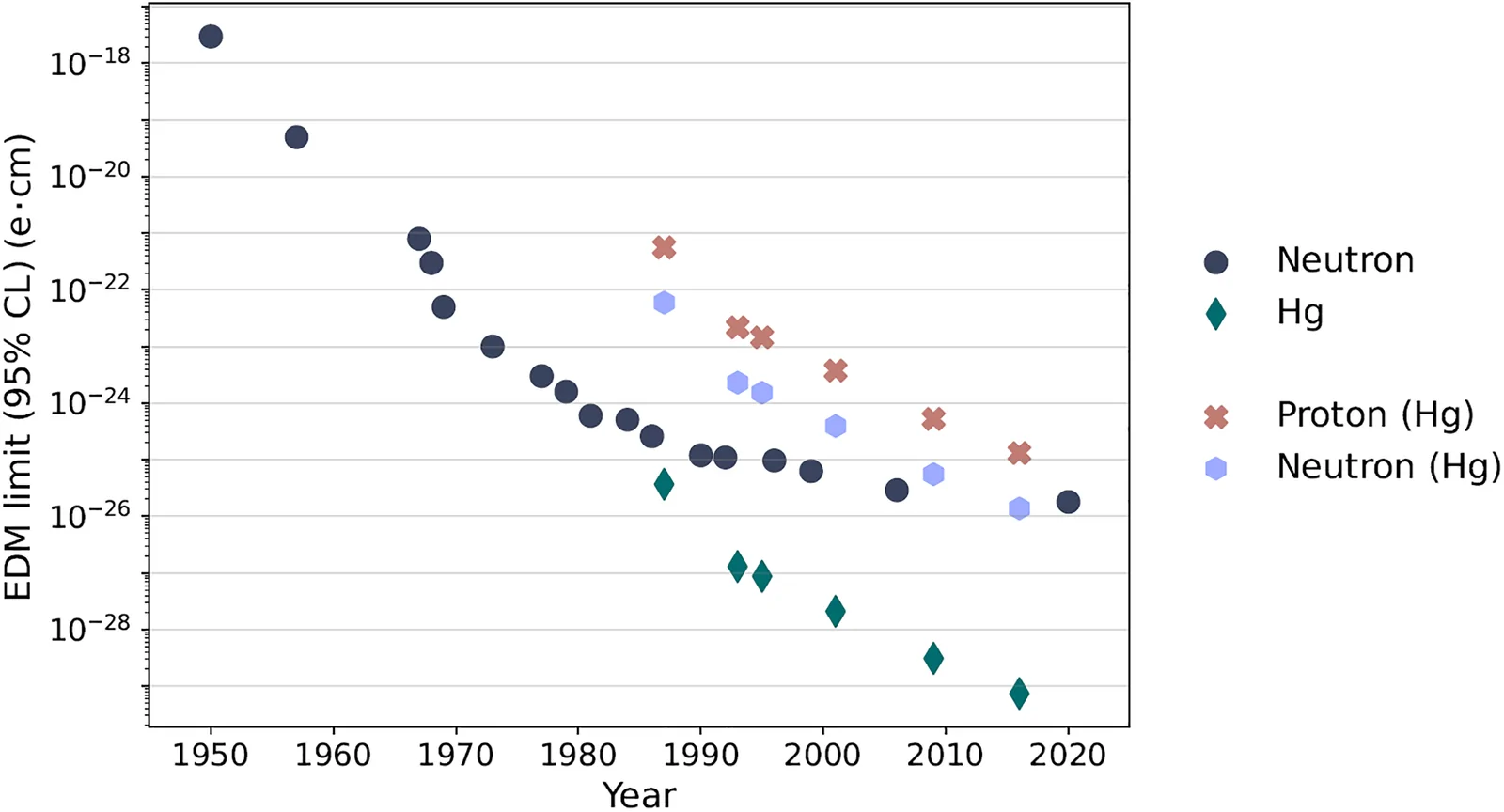
EDM limits. The history of direct limits on the neutron EDM from the first experiment (*53*) until today at $2\times 10^{-26}$ e⋅cm (*115*) along with the EDM limits for the ^199^Hg atom, providing the most precise directly measured limit of any EDM today at $7.4\times 10^{-30}$ e⋅cm (*116*, *117*). From the ^199^Hg measurement, one can, with some disputed large uncertainties and some suppression factor, also derive indirect limits on the neutron and proton EDMs (*118*), displayed without uncertainties. The direct and indirect neutron limits are about equal; for the proton, no direct limit is available yet. Figure credit: T. Hume/*PSI*.

Ramsey himself later often mentioned that although they performed the experiment in the early 1950s, they were discouraged from quickly publishing by the strong belief in parity conservation. As this belief broke down with the experiments of Wu, Ambler and colleagues (*50*) studying β decay of spin-polarized ^60^Co nuclei in 1956 (see Fig. 4), and subsequent muon decay experiments of Garwin *et al.* (*51*) and Friedman and Telegdi (*52*), the first direct experimental limit of the neutron EDM was published by Smith *et al.* (*53*). We note the promotion of the PhD student from an acknowledgment to being the first author on the paper. Incidentally, Smith was later an undergraduate physics instructor of one of us (W.M.S.) at the University of Illinois. With the possibility of symmetry violations, at least in weak interactions, now being beyond esoteric ideas, Ramsey (*54*) also published in 1957 on the possibility of time-reversal violation and the need for experimental tests against theoretical bias. Here again, the neutron EDM was the first choice to be pursued and has been among the most sensitive probes since [presently, several international collaboration are working toward one to two orders of magnitude improvements in sensitivity of neutron EDM measurements (*55*); see Fig. 5]. Immediately after the publication of the first neutron result, still in 1958, a muon EDM measurement was carried out (*56*), as in the previously mentioned parity violation studies, benefiting from polarized muons obtained in pion decay and polarization analysis due to the muon decay asymmetry. Already mentioned in (*56*) from a private communication with Feinberg (*57*) is an indirect limit of order 10^−13^ e⋅cm for the electron, estimated from the Lamb shift in hydrogen. The work by Feinberg was submitted on the same day as the muon result, but it was published later, back to back with a similar result from Salpeter (*58*).

### The story of ultracold neutrons and exotic spin–dependent interactions

Not long after Chadwick’s 1932 discovery (*59*) of the electrically neutral neutron, a spin-1/2 particle such as the proton, it was shown that the neutron had a magnetic moment of a size similar to that of the proton. Along with the strong deviation of both the neutron and proton gyromagnetic ratios away from the value of 2 expected in Dirac’s relativistic theory of the electron, this was a strong hint that neutrons and protons both probably had an internal structure of some type. Decades later, electron scattering experiments lastly resolved the quarks whose dynamical motion and intrinsic magnetic moments combine to generate the magnetic properties of the neutron and proton [see, for example, (*60*)]. In the past few decades, the scientific mystery has shifted from the magnetic moments back to the spins of the neutron and proton: Where exactly does the spin come from? The most obvious guess, namely, that two of the three spin-1/2 quarks inside the proton and neutron simply pair, is wrong experimentally. We now know that a large fraction of the proton and neutron spin comes from the electrically neutral gluon field, which seems to confine the quarks inside protons and neutrons. The scientific lesson is clear: One can learn about fundamental questions by paying close attention to spin and the dynamical properties associated with it.

Soon after neutron beams from nuclear reactors became available for study in the late 1940s, scientists were quick to take advantage of the special combination of properties had by these slow neutron beams. These beams are electrically neutral, but just magnetic enough for the spin to be manipulated delicately: able to penetrate macroscopic amounts of matter, while still interacting coherently.

Fermi and Zinn (*61*) showed that slow neutrons can experience total external reflection from flat surfaces, in contrast to the internal reflection of light in most media, thereby initiating a key technology for slow neutron manipulation. Zel’dovich (*62*) later noted that one can confine very-low-energy neutrons, now called “ultracold” neutrons (UCNs) inside material bottles. Shapiro (*63*) suggested the use of polarized UCNs to search for the neutron EDM and led one of the first experiments (*64*), which, in parallel with Steyerl’s work (*65*), successfully extracted and measured UCNs. Now, spin-polarized UCN ensembles are created nearly at rest in the laboratory to extend the measurement time for EDM and neutron β decay measurements. UCNs are created not by laser cooling as done for atoms (since there are no neutron-electron bound states) but by phonon cooling via neutron creation of phonons in a cold, dense medium from which the cooled neutrons can escape and be guided through tubes to the measurement chamber.

### Reasons for measuring EDMs

As discussed in the “Magnetic and electric dipole moments in the Dirac equation” and “EDM history” sections, EDMs violate parity and time-reversal symmetries. If physical reality is described by a quantum field theory, then the *CPT* theorem (the *C* refers to charge conjugation) guarantees *CPT* invariance based on most fundamental assumptions (*66*–*70*). The *T*-violating character of EDM therefore immediately corresponds to *CP* violation, with *CP* being the symmetry connecting matter and antimatter. It turns out that the sensitive searches for finite permanent EDM present crucial tests of *CP* invariance, and the absence of signals so far provides some of the most constraining bounds on models and theories of *CP* violation BSM of particle physics. A framework for incorporating the experimental upper limits in several systems into BSM physics is provided by effective field theory (EFT) combined with the sensitivity of each system to EFT parameters (Wilson coefficients) provided by atomic and nuclear theory (*71*). With this input, a global analysis to constrain BSM physics was introduced (*72*) and continues to be refined (*73*).

As the SM is believed to not provide sufficient amounts of *CP* violation to explain the observed baryon asymmetry of the universe (*74*, *75*), many such BSM models are motivated by the need to provide additional sources of *CP* violation. In general, many BSM theories naturally introduce *CP* violating parameters constrained only by experimental results. The known *CP* violation of the EW SM induces finite EDM, which is, however, still many orders of magnitude smaller than experimental sensitivities. In addition, the strong interaction of the SM allows for a *CP* violating phase, which is, however, already severely constrained by experimental limits on the neutron and nuclear EDM to be small of order of at most 10^−10^ or zero, constituting the so-called “strong *CP* problem.” For more extensive overviews, see, for example, (*76*, *77*).

**Fig. 6.**
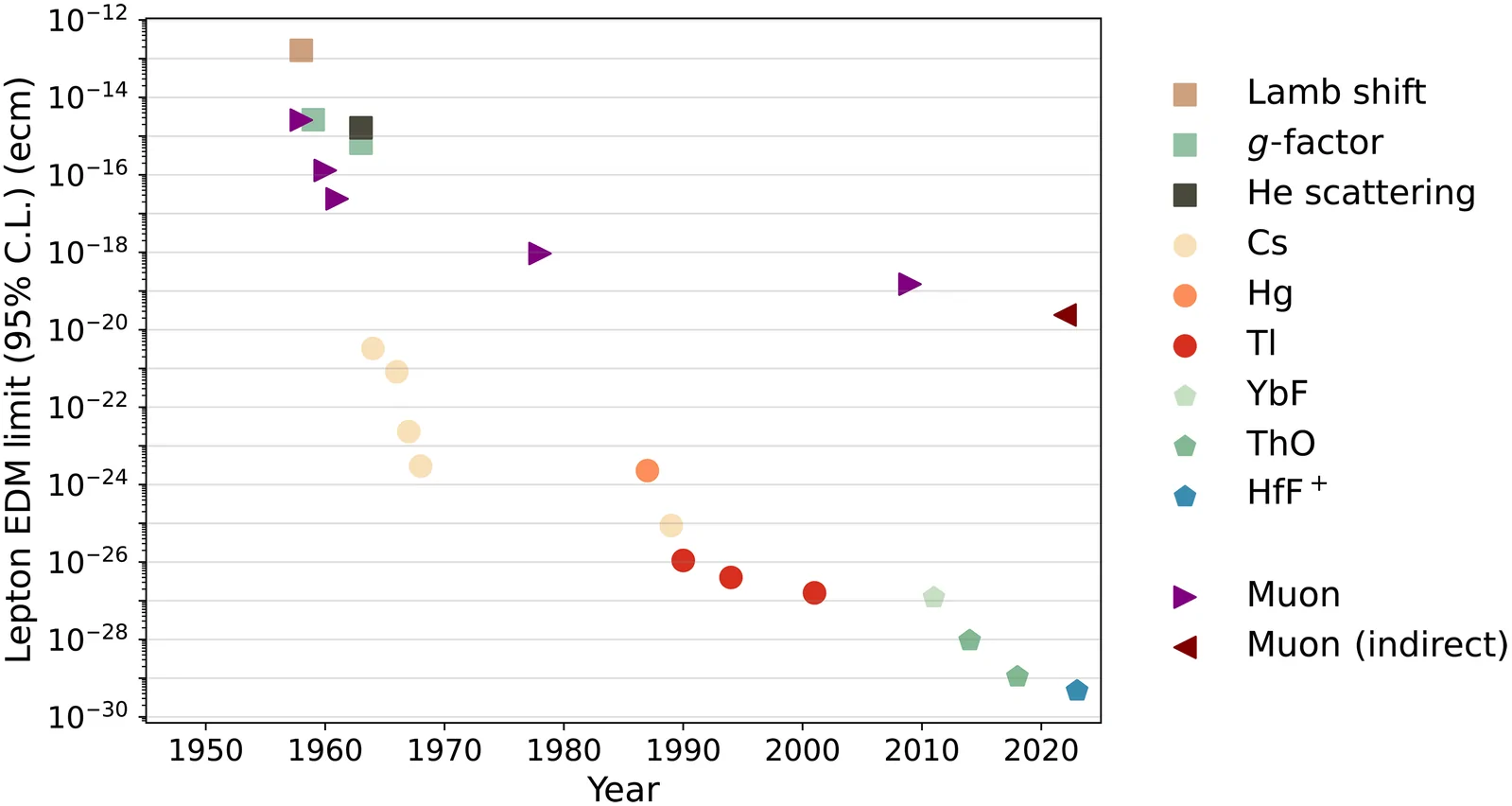
Electron and muon EDM limits. The history of electron EDM limits, indirectly derived from various systems. The most sensitive measurements today use molecules, the best one on the HfF^+^ ion setting a limit of $5\times 10^{-30}$e⋅cm for the electron (*119*). Opposite to the case of the diamagnetic ^199^Hg atom with its suppression factor (see Fig. 5), the paramagnetic atoms and molecules exhibit large amplification factors. Also shown are the limits on the muon EDM, the best one published today still coming from the BNL muon *g* − 2 experiment (*120*). The muon is the only measurement on a bare fundamental particle. Figure credit: T. Hume/*PSI*.

Over the years, the field of EDM searches has become much more diverse compared to the earlier work (see the “EDM history” section), and, in particular, atoms and molecules entered the arena, allowing for extremely sensitive measurements of the electron and nuclear EDMs due to large possible amplification factors (see Fig. 6). A review of the present status of the field is beyond the scope of this article; however, we note that there is now a vibrant community and a plethora of approaches to searches for permanent EDMs (*55*), including initially unexpected directions, such as the use of polyatomic molecules and storage rings as high-precision traps for charged particles.

## EXOTIC SPIN–DEPENDENT INTERACTIONS

### Reasons for exotic interactions

In the same marriage of relativity and quantum mechanics from which spin naturally emerges also comes the demand that interactions between particles be mediated locally through the exchange of a force carrier. As for all particles, the force carrier must have mass (which can be zero, as for the photon and graviton) and spin, and different forces have different amplitudes per unit spacetime volume (which we call charges or couplings) to emit and absorb the associated force carrier. The basic structure of the interactions in the nongravitational component of our present Standard Model of particles and interactions emerges if one labels the force associated with one type of charge electromagnetic, the force associated with two types of charge that can transform into each other weak, and the force associated with three types of charge that can transform among each other strong. Combined with the demand that the probability for any process in quantum mechanics cannot be greater than one, which leads to the conservation of the associated charge, most of the general features of the so-called strong, electromagnetic, and weak interactions emerge. Since the fundamental constituents of matter we know of are all spin-1/2 particles, the spin of the force carrier in electromagnetism, the photon, must be at least spin 1 (otherwise, like charges in electrostatics would attract rather than repel), and, likewise, the *W* and *Z* bosons of the weak interaction and the gluons of the strong interaction are also spin 1. These interactions are implemented mathematically through the gauge field principle and result in renormalizable quantum field theories. Once again, we see that spin dictates many features of the forces we see in nature.

It is always possible that new weak forces await discovery. However, what makes a force weak? It is not just the strength of the charge that matters: Even if the charge is strong, if the spatial range of the force carrier is small because of its large mass, then the force never gets anywhere. This leads to two different types of weak forces: One type has large charges and large mass for the force carrier, and the other has small charges and small mass for the force carrier. The first option attracted the most attention after the Standard Model was established as it is easier to make consistent with the idea of the unification of the three nongravitational forces into one. Possible new short-range interactions from new heavy particles are now classified using EFT techniques.

With time and with the discovery of dark matter (DM) and dark energy, the second option of new weakly, coupled longer-range interactions is being taken more seriously. Fortunately, the infinite number of possibilities one might think this idea would lead to are still tightly constrained if one adheres to the same ideas of relativity and quantum mechanics that give rise to spin. For this reason, there has been an explosion of experimental searches for weakly coupled interactions mediated by light particle exchange, and weak spin–dependent interactions are natural candidates for forces that might have been missed in previous experiments.

### History of the exotic potentials

All of the known forces except gravity have spin-dependent components to their interactions that lead to qualitatively distinct physical effects, which have been clearly identified in experiments. However, there is a vast unexplored range in coupling strength and exchange boson mass of possible exotic spin–dependent interactions that could exist and a comparably wide variety of theoretical speculations that can produce exotic spin–dependent interactions as by-products of their attempts to explain various mysteries unaddressed in the Standard Model. This situation has motivated an explosion of experimental work to search for exotic spin–dependent effects over the past couple of decades.

In particular, sensitive searches for these interactions can be conducted among the protons, neutrons, and electrons, which compose normal matter, if one can design experiments and environmental conditions that suppress the background effects from the known interactions. Recently, this program has also been extended to purely antimatter systems, where antihydrogen spectroscopy enables tests of exotic interactions between positrons and antiprotons (*78*). Since spin in Standard Model particles is accompanied by ordinary magnetic moments, exotic spin–dependent interactions are often difficult to disentangle from conventional magnetic effects. In experiments using polarized electrons, nuclei, or macroscopic spin ensembles, magnetic dipole interactions, field gradients, and environmental magnetic noise can mimic or mask a new force. Although magnetic backgrounds are not necessarily, the dominant limitation in all approaches, for example, in some atomic and molecular parity violation experiments (*79*), their control or rejection remains a central issue in many spin-based searches, including electric dipole moment experiments, as discussed in the “Electric Dipole Moments” section.

Since a large fraction of the experiments that have been conducted in this field use matter in nonrelativistic motion in the laboratory and since the effects are weak enough that they can be evaluated in the limit of single spin 0 or spin 1 boson exchange, it is convenient to classify the possibilities by taking the nonrelativistic limit of single boson exchange for all of the possible allowed types of couplings that can be present in the fermion currents (scalar, pseudo-scalar, vector, axial vector, and tensor) of the two interacting particles. This exercise generates a finite set of potentials that depend on the spins and velocities of the interacting particles. A recent review of this field (*80*) presents the current experimental constraints that come from a variety of experimental probes: torsion balances with polarized test masses and ensembles of optically pumped atoms, which usually explore force ranges of macroscopic scale, NV centers in diamond and polarized slow neutron spin rotation, which probe mesoscopic distance scales, and analyses of precision measurements in atomic physics to search for angstrom-scale interactions.

The special properties of slow neutrons allow them to also probe even more fundamental aspects of spin. The wave function of a spin-1/2 particle in quantum mechanics is predicted to change sign under a 2π rotation: One must rotate the particle by 4π to return it back to the same state. One can test this prediction by passing a spin-1/2 particle into an interferometer, rotating the spin in one of the paths by an adjustable angle relative to that of the other path, and observing the interference pattern upon recombination. In the mid-1970s, Rauch *et al.* (*81*) and Werner *et al.* (*82*) developed a neutron interferometer using dynamical diffraction from cutout blades of a monolithic crystal of silicon. The capability of this device to separate the two paths of the neutron by macroscopic distances enabled precisely this experiment, with the relative rotation of the neutron spin on the two interferometer paths performed by magnetic fields, which can precess the magnetic moment (and, therefore, the spin tied to it) by an adjustable angle. Their clear demonstration (*81*, *82*) of the minus spin of the neutron spin under a 2π rotation is an example of one of the beautiful measurements, testing and illustrating fundamental principles of quantum mechanics that this technology enabled.

The minus sign of a spin-1/2 particle under a 2π rotation is consistent with the minus sign one gets in the exchange of two spin-1/2 particles from Fermi statistics, since the exchange of two identical particles is equivalent to a relative rotation of the two particles by 2π. However, this minus sign is known to rely on the three dimensionality of rotations in space: In cases where the system dynamics are constrained to two dimensions, one can, in principle, get any relative phase factor one wants under the interchange of two identical particles. The so-called “anyons,” which live in such a two-dimensional (2D) world (*83*–*85*), can be realized in condensed matter and have dynamics that make them interesting candidates for future applications in quantum information.

In 4D spacetime, the rotational invariance of space generalizes to 4D combinations of rotations and boosts. The *CPT* transformation can be viewed as a (complex) rotation in 4D spacetime (*86*). The connection between spin and statistics (*87*, *88*) is not an independent ingredient in the *CPT* theorem: It follows already from two of the assumptions (energy positivity and microscopic causality) also needed in the *CPT* proof. What happens if *CPT* is violated? In this case, the spin statistics connection can survive, but Lorentz symmetry must be violated (*89*). This latter result forms the foundation for a wide variety of experimental searches for *CPT*/Lorentz violation [see, for example, review (*90*)].

### Spin and gravity

In view of the intimate connections between the properties of spin and the nature of spacetime, it is expected that speculations about possible connections between spin and spacetime geometry are a recurring theme in theoretical physics. Ever since the geometric description of gravity developed by Einstein, which connected mass to spacetime curvature, there has long been a theoretical suspicion of a similar relationship of spin to spacetime geometry. Wigner’s analysis (*91*) of the quantum states of free point particles in relativistic spacetime in terms of irreducible unitary representations of the Poincare group concludes that all these particles can be classified by their mass and spin. It is also an interesting geometrical fact that, in 4D metric spacetime, there are two independent tensor structures characterizing the space that can appear, namely, curvature and torsion. Is this match of the two properties of point particles and the two flavors of tensors in 4D curved spacetime geometry just a coincidence? If, in addition, one adds the locality requirement for symmetries and elevates the entire Poincare symmetry to a local symmetry, then one naturally gets a generalization of Einstein’s theory called Einstein-Cartan gravity in which mass generates spacetime curvature and spin generates torsion (*92*, *93*).

Spin-dependent gravitational phenomena are already a part of standard general relativity through what one might think of as “gravitomagnetic” effects. Much like the magnetic moment dynamics of a particle moving in a magnetic field, the angular motion of particles in general relativity should also exhibit these effects, with contributions from both the orbital motion proportional to $\vec{L}$ and the spin contribution proportional to $\vec{s}$. The legendary Gravity Probe B satellite resolved gravitomagnetic effects proportional to $\vec{L}$ in the curved spacetime of Earth, but, so far, no one has yet seen the associated spin effect, and the Gravity Probe Spin idea (*94*) is named in its honor.

## SPIN, DM, AND GRAVITATIONAL WAVES

The origin of DM apparently constituting some 80% of all matter in the universe remains unknown. It may therefore be unexpected that we can rather confidently state that if galactic DM consists primarily of particles of a certain kind and if the mass of the particle is less than a few electron volts, then the spin of this DM particle should be integer. However, how can we know a spin property of an unknown particle? The argument goes like this. We suppose that the particle has a half-integer spin. Then, according to the spin statistics theorem, these particles are fermions. Now, the gravitational potential of a galaxy can be thought of as a “box” containing the DM particles, which assume a Fermi-Dirac distribution if the particles are fermions. For our galaxy, we know the local DM density to be about 0.4 GeV/cm^3^, while the escape velocity from the galaxy is on the order of $10^{-3}c$. A quick back-of-the-envelope estimate shows that for a gas of particles lighter than a few electron volts, the Fermi velocity exceeds the galactic escape velocity and because DM is associated with the galaxy and does not “fly away,” the particles cannot be fermions and must instead be bosons, i.e., have integer spin.

When it comes to the search for ultralight DM, spins play a central role as the core element of many detectors. This topic has been recently reviewed in (*95*), with particularly sensitive detectors based on a mixture of alkali-metal and noble-gas spins discussed in (*96*). A recent realization is that modern spin–based sensors may also be sensitive to gravitational waves [see (*97*) and references therein].

## BIG EFFECTS OF A “SMALL” SPIN

The scale of elementary particle spin, $\hbar \approx 10^{-34}\;J\cdot s$, is minuscule compared to the angular momenta that we encounter in daily life. However, this in no way means that the effects of spin are small or even subtle.

To start with, the entire structure of chemistry is governed by the fact that electrons are fermions. Should electrons have been bosons, the ground state of all atoms would have been $1s^{Z}$, where *Z* is the atomic number, and there would have been nothing like a periodic table of elements. Incidentally, the “boring” bosonic atoms might exist in nature. If there exist beyond-standard bosons like, for example, axions, then they could accumulate around gravitating objects such as stars or planets just in such form (*98*).

We have already discussed in the “Proton spin” section the example of parahydrogen, which shows that nuclear spin properties govern the rotational states of dimer molecules, thus controlling energy scales that markedly exceed those of direct spin-spin interaction.

An example at the interface of atomic and particle physics is positronium, the bound state of an electron and a positron. In this exotic form of “hydrogen,” the ground state is 1*S*, but the total spin can be either *J* = 0 (parapositronium) or *J* = 1 (orthopositronium), depending on how the spins of the electron and positron add together. Again, the difference in the spin arrangement has marked effects on the atom. For example, the lifetime of orthopositronium (142 ns) is three orders of magnitude longer than that of parapositronium (125 ps).

Spin is already a major factor in things that have not even been found or whose origin and composition are yet to be understood. An example is a model where galactic DM consists of one type of particle with a mass less than a few electron volts that only interacts via gravitational forces (*99*), as discussed in the “Spin, DM, and Gravitational Waves” section. The list of giant effects of small spin can be continued, for example, with neutron stars and supernovae, but we stop here, hopefully, having already made the important point.

## SPINS IN SOLIDS AND LEVITATED MAGNETS

One of the most notable and consequential manifestations of electron spin is ferromagnetism. Through exchange interactions, spins in a ferromagnetic domain (e.g., in a solid) lock to each other and point in a common direction. The coupling of the overall spin resulting from the contribution of a large number of atomic spins with macroscopic motion of the magnet provides a powerful way to probe spin physics. This goes back to the discovery by Einstein and de Haas around 1915 (*100*), i.e., before the discovery of spin, that a suspended stationary magnet starts to rotate when it is brought above the Curie temperature and the magnet depolarizes. (Note the instructive example of a theorist coauthoring an experimental paper.) The inverse effect of magnetization being induced by rotation was found by Barnett at about the same time (*101*). The corresponding effects for nuclear spins are also of great interest and may be useful, for example, for polarizing nuclei for magnetic resonance experiments (*102*).

We mention here a recent twist on this story. If you put a spin in a magnetic field, then it precesses around the direction of the field. If you do the same with a free-floating ferromagnetic (e.g., compass) needle, then a completely different, oscillatory motion in a plane containing the field is usually observed. However, it was pointed out (*103*) and then confirmed by detailed theoretical calculations (*104*) that a free-floating ferromagnetic needle should also precess if the magnetic field is sufficiently weak. Recently, the effect was demonstrated experimentally (*105*). Levitated magnets have also been shown to be excellent sensors (for example, of magnetic or exotic pseudomagnetic fields), and they are currently being developed for searches for ultralight DM (*106*) and detection of the general relativity effects on spins (*94*).

## CONCLUSION

We hope that this brief article sufficiently illustrates the key role that spin has played in fundamental physics in the 100 or so years since its discovery and may play in the years to come. The limited scope has not allowed us to elaborate on many important subfields of spin physics. For example, in the “The story of ultracold neutrons and exotic spin–dependent interactions” section, we have discussed that confining (trapping) ultracold neutrons in “bottles” represents a key enabling technology for studying fundamental spin properties such as EDMs. However, more generally, confining spins plays a key role across the spectrum of fundamental experiments and applications of spins. Examples include the use of Teflon-coated storage cells for polarized hydrogen in hydrogen masers (*107*), paraffin-coated cells used in atomic magnetometers (*108*), laser-cooled and trapped atoms and molecules with spin, charged particles of matter and antimatter, and atomic and molecular ions with spin confined in Paul or Penning traps or storage rings, etc. Spin is also central to precise and sensitive measurements; for example, magnetometry, which is often an indispensable part of fundamental experiments (*109*).

As spin is clearly a cornerstone of modern physics and because physics is fundamentally an experimental science, it is essential to subject the foundations of physics to unrelenting experimental scrutiny. This is true even without a specific theoretical basis for a possible violation of the laws. Examples of this include tests of spin statistics connection, Pauli Exclusion Principle for fermions, Bose-Einstein statistics for bosons, and tests of *CPT* and Lorentz Invariance (*90*, *110*, *111*). We look forward to the essential role spin that will continue to play in addressing the mysteries of our universe and probing the most fundamental principles of physics.
